# Integrating HIV care into nurse-led primary health care services in South Africa: a synthesis of three linked qualitative studies

**DOI:** 10.1186/1472-6963-13-171

**Published:** 2013-05-07

**Authors:** Kerry Uebel, Andy Guise, Daniella Georgeu, Christopher Colvin, Simon Lewin

**Affiliations:** 1 Department of Internal Medicine, Faculty of Health Sciences, University of the Free State, Nelson Mandela Drive, Park West, Bloemfontein 9301, South Africa; 2 Free State Department of Health, Charles Street, Bloemfontein 9301, South Africa; 3 London School of Tropical Hygiene and Medicine, Keppel Street, London WC1E 7HT, United Kingdom; 4 Knowledge Translation Unit, University of Cape Town Lung Institute, University of Cape Town, George Street, Mowbray, 7700, Cape Town, South Africa; 5 School of Public Health and Family Medicine, Falmouth Building, Faculty of Health Sciences, University of Cape Town, Observatory, 7925, Cape Town, South Africa; 6 Norwegian Knowledge Centre for Health Services, Pilestredet Park 7, 0176, Oslo, Norway; 7 Health Systems Research Unit, Medical Research Council, Francie van Zijl Drive, Tygerberg, 7505, Cape Town, South Africa

**Keywords:** Integration, HIV care, Primary health care, Nurse specialisation, Stigma

## Abstract

**Background:**

The integration of HIV care into primary care services is one of the strategies proposed to increase access to treatment for people living with HIV/AIDS in high HIV burden countries. However, how best to do this is poorly understood. This study documents different factors influencing models of integration within clinics.

**Methods:**

Using methods based on the meta-ethnographic approach, we synthesised the findings from three qualitative studies of the factors that influenced integration of HIV care into all consultations in primary care. The studies were conducted amongst staff and patients in South Africa during a randomised trial of nurse initiation of antiretroviral therapy (ART) and integration of HIV care into primary care services – the Streamlining Tasks and Roles to Expand Treatment and Care for HIV (STRETCH) trial. Themes from each study were identified and translated into each other to develop categories and sub-categories and then to inform higher level interpretations of the synthesised data.

**Results:**

Clinics varied as to how HIV care was integrated. Existing administration systems, workload and support staff shortages tended to hinder integration. Nurses’ wanted to be involved in providing HIV care and yet also expressed preferences for developing expertise in certain areas and for establishing good nurse patient relationships by specialising in certain services. Patients, in turn, were concerned about the stigma of separate HIV services and yet preferred to be seen by nurses with expertise in HIV care. These factors had conflicting effects on efforts to integrate HIV care.

**Conclusion:**

Local clinic factors and nurse and patient preferences in relation to care delivery should be taken into account in programmes to integrate HIV care into primary care services. The integration of medical records, monitoring and reporting systems would support clinic based efforts to integrate HIV care into primary care services.

## Background

Despite substantial international progress towards achieving universal access to antiretroviral therapy (ART) for people living with human immunodeficiency virus (HIV), it was estimated that, by the end of 2010, only 47% of the 14.2 million people eligible for ART were receiving it [1]. Strategies to improve access to ART in low and middle income countries with health system constraints include task-shifting [2] and community mobilisation [3,4] as well as integration of HIV care, including the provision of ART, into primary care services in ways that strengthen health systems [5,6].

The integration of HIV care into primary care services is also seen as an important strategy to provide coordinated care for HIV/AIDS and other related health needs such as tuberculosis (TB) [7,8] and sexual health [9] and to generally support the provision of holistic care and counter the fragmentation that characterises single disease programmes [10]. This is not unique to HIV care: there have also been calls to integrate sexual and reproductive health [11] and mental health care [12] into primary care services. The debate on the merits of integrated compared to single disease approaches to primary care (the so-called horizontal versus vertical approach) has continued for many years [13,14], yet there are still few clear recommendations on the extent of integration of services that is needed or useful in primary care. Although the concept may be intuitively appealing, there is a lack of strong evidence that integration of services leads to improved health outcomes and therefore a need exists for more studies on the effectiveness of integrated health programmes [15].

One of the reasons behind the lack of clear guidelines and evidence is that integration is a very broad concept. It has been defined as “a variety of managerial or operational changes to health systems to bring together inputs, delivery, management and organisation of particular service functions” [13]. It can involve various health system functions including governance, planning, financing, monitoring and evaluation and service delivery [16]. In practice, there is wide organisational variety across different health programmes internationally and very few are fully integrated in all of these areas of health system functioning [17]. Given the broad nature of integration, any studies of the outcomes of programmes to integrate health services need to document clearly the levels of health system functioning that are being integrated so that evidence of outcomes can be compared across different interventions [17].

A range of strategies have been described in the specific area of the integration of HIV care into primary care services in South Africa and other low and middle income countries. These strategies have included co-location of vertically run HIV services within the same facilities as primary care services [18]; co-provision of ART within home based directly observed TB treatment programmes [19]; down referral of patients stabilised on ART from hospital-based ART clinics to primary care clinics [20,21]; and the provision of outreach support to primary care clinics from existing ART sites [22]. Other programmes have described strategies to integrate HIV care into all primary care consultations, for example through staff training, standardised protocols, combined waiting areas and medical records, and the inclusion of HIV testing in triage [7,23]. These reports have not commented on the degree of integration achieved in primary care, particularly within clinics, or on the barriers and facilitators to integration of HIV care.

This article responds to the need to document interventions to integrate HIV care into primary care services, to record outcomes of these interventions and to provide analytical insights to inform both programme management and further theoretical development of the area. We report on factors perceived to influence the integration of HIV care into primary care services at the level of service delivery during a randomised controlled trial of strategies to improve access to ART in South Africa: the Streamlining Tasks and Roles to Expand Treatment and Care for HIV (STRETCH) trial [24]. Lessons learnt in the area of integration of HIV care may be useful in informing broader questions regarding the integration of programmes into service delivery in primary care.

### Context: the Free State ART programme and the STRETCH trial

The Free State province of South Africa has an estimated HIV prevalence of 18.5% amongst 15–49 year olds [25]. The public sector ART rollout commenced in 2004 with a vertical approach to the delivery of HIV care. Patients diagnosed as HIV positive in primary care clinics were referred to ART nurses in ART assessment sites located within certain existing provincial primary care clinics for all further HIV care. Patients not yet eligible for ART were seen at 6–12 month intervals at these assessment sites for CD4 counts, staging and TB screening. Patients eligible for ART (CD4 < 200 or Stage 4 AIDS) accessed drug readiness training, baseline blood tests and monthly ART supplies at these assessment sites, but were referred to accredited ART treatment sites within designated hospitals for doctors to initiate and repeat ART prescriptions. By mid-2007, 57 ART assessment and treatment sites were functioning [26] but less than a quarter of the primary care clinics in the province were able to offer on-site access to ART services. By mid 2008, only an estimated 25% of those needing ART were receiving it in the Free State and this was the lowest coverage in South Africa with national coverage estimated at 40% [27].

The STRETCH trial, a pragmatic, cluster randomised, controlled trial, was conducted in the Free State from 2007 to 2010, to assess strategies to improve access to ART. Thirty one ART assessment sites were randomised into 16 intervention and 15 control sites [24]. The trial comprised two main interventions: nurse initiation and repeat prescription of ART for adults with uncomplicated HIV; and integration of HIV care into primary care services. Two salient features of the implementation of the integration interventions were: the role of the trial manager (KU) as an agent of change, and the involvement of local clinic staff in the development and implementation of the integration intervention [28].

The integration intervention that was developed and the existing system for delivering HIV care are compared in Table 1. Three elements of pre-ART care – voluntary counselling and testing (VCT); initial CD4 counts; and 6-monthly routine HIV care – as well as three elements of ART care – baseline blood tests; drug readiness training; and monthly supply of ARVs – were to be integrated into primary care services at two different levels. The first level was integrating pre-ART and ART care into the routine consultations of all nurses within the intervention clinics (internal integration). The second level was to enable other primary care clinics, which before the trial were referring all their HIV positive patients to intervention clinics, to provide pre-ART and ART care to their own patients (mainstreaming HIV care). During the trial, training in HIV care was being rolled out to all primary care nurses but training and authorisation for nurses to initiate and repeat six month prescriptions of ARVs was limited to nurses at intervention ART sites only.

**Table 1:** Roles of primary care clinics with and without ART assessment sites in the delivery of elements of HIV care before and during the STRETCH intervention

	**Primary care clinics without accredited ART site**	**Primary care clinics with accredited ART assessment site functioning within the clinic**
**Organisation of HIV care before the STRETCH trial**		
*Delivery of pre-ART care (voluntary counselling and testing, initial CD4, 6–12 monthly HIV care) and ART care (baseline bloods, drug readiness training and monthly issuing of ARVs)*	• Primary care nurses identify and refer HIV positive patients to ART nurses at their referral primary care clinic with an accredited ART assessment site	• Primary care nurses identify and refer HIV positive patients to ART nurses working within that clinic
		• ART nurses provide pre-ART and ART care for all patients referred from primary care services
		• All patients needing ART initiation and re-prescription referred by ART nurses to doctors at ART treatment sites
**Organisation of HIV care during the STRETCH trial (intervention clinics and their referring primary care clinics only)**		
*Delivery of pre-ART care (voluntary counselling and testing, initial CD4, 6–12 monthly HIV care)*	“**Mainstreaming pre-ART care”**	**“Internal integration of pre-ART care’**
	• Primary care clinic enabled to provide pre-ART care to their own patients	• All professional nurses (ART and primary care nurses) at intervention clinic encouraged to provide pre-ART care as part of routine consultations
*Delivery of ART care (baseline bloods, drug readiness training and monthly issuing of ARVs)*	**“Mainstreaming ART care”**	**“Internal integration of ART care”**
	• Primary care clinic enabled to provide ART care to their own patients	• All professional nurses (ART and primary care nurses) at intervention clinic encouraged to provide ART care as part of routine consultations
*Initiation and re-prescription of ART during STRETCH intervention*	• All patients needing an ART prescription referred to ART nurses at intervention clinic	• All professional nurses (ART and primary care nurses) at intervention ART sites trained and authorised to initiate and repeat ART prescriptions
		• Complicated patients referred to doctor at ART treatment site

An integration questionnaire was developed and administered at intervals at all the clinics involved in the STRETCH trial in order to assess progress of the integration intervention. This assessment showed that there was significant progress made in the mainstreaming of HIV care but no significant progress made with internal integration [29]. The factors that may have contributed to this unexpected lack of progress in internal integration within intervention clinics in the STRETCH trial are the focus of this article. We report a synthesis of the findings of three qualitative studies conducted in the Free State during the time period of the STRETCH trial. This synthesis was undertaken in order to capture a broad picture of the factors that may have impacted on the integration of HIV care into all consultations in primary care.

## Methods

In this paper we synthesise the findings from three separate but linked qualitative studies. Two were conducted as a part of a process evaluation of the STRETCH trial (KU, DG) [30] and the third explored primary care experiences of integration of HIV care in several primary care clinics (AG) [31], some of which were part of the STRETCH trial. The studies led by DG and AG included interviews and focus group discussions conducted within clinics with patients and staff from all services not only the ART services. This data collection was in English for the majority of staff and in a local language, seSotho, for patients and some staff, using interpreters. Interviews and focus groups followed a semi-structured approach, with efforts to explore specific themes but also responding to issues raised by respondents. Interview recordings were transcribed and analysed thematically. KU and AG also conducted semi-structured observations, focused on understanding clinic activity around patient care, and participated in clinic meetings and other everyday interactions with staff and patients. Each study addressed different but related research questions, and each provides a rich account of how the integration of HIV care was experienced and implemented in primary care within the context of the STRETCH trial. Table 2 outlines the different focus, methods and data collected for each study. Although each study was conducted separately, there were common institutional links through the University of Cape Town and the investigators discussed these studies regularly during their implementation.

**Table 2:** Summary of the methodology used in the three qualitative studies

**Study focus**	**Study approach**	**Data collection method relevant for this synthesis**	**Detail of data collection**
Study 1: STRETCH trial – trial of intervention to integrate HIV care into PHC and task shifting of initiation and prescription of ART to nurses	Randomised controlled trial, with participant observation by trial manager (KU)	Participant observation	170 visits of approximately two hours each to 31 clinics, conducted over four years while managing the trial; notes of visits kept in a diary
Study 2: STRETCH process evaluation – the evaluation explored all aspects of the intervention (training, managerial issues etc.) including issues of HIV care integration	Mixed-method qualitative evaluation (led by DG with SL and CC)	Focus group discussions	10 focus groups with nurses 6 focus groups with patients
		In-depth and key Informant Interviews	26 interviews with facility managers, doctors, trial manager, local/district/provincial health managers and key stakeholders
		Observation	7 observations of support workshops for nurse-trainers and trial manager support visits to clinics
Study 3: Qualitative study to understand the organisation of PHC, following the integration of HIV care and task shifting in PHC shifting.	Mixed-methods study, based on ethnographic principles (AG, supervised by SL)	Observation	Observation in 4 clinics, including 2 STRETCH trial clinics, over a 15 month period. Emerging themes were explored in an additional 6 clinics, including 3 STRETCH trial clinics.
		Interviews	Interviews with 34 professional nurses, 6 other members of clinic staff and 21 patients

Insights from the three studies were combined based on the meta-ethnographic approach to synthesising qualitative data from published studies by different researchers in different settings [32]. We did not adhere to all seven steps of the meta-ethnographic approach as originally set out by Noblit and Hare and cited by Britten et al [33,34]. The first three steps of getting started, identifying focus of interest and then identifying and reading relevant studies are suitable for the synthesis of data from published qualitative studies identified as part of a review. For this study, where the focus and studies were already identified at the start, we based our synthesis around the final four steps of this approach: 1) identifying relations between the studies, 2) translating them in to one another, 3) synthesising these translations into higher order interpretations and then, lastly, 4) communicating the findings [33]. We consider that our approach conformed to the original intention of the meta-ethnographic approach through preserving the relationships between concepts or themes identified by the primary studies while facilitating comparison across the studies.

The process began with initial, unstructured discussion amongst the authors regarding the key issues identified by each study and the extent to which it would be useful to attempt to synthesise the findings of the three studies. KU, DG and AG then held a structured meeting during which the primary (participant understanding) and secondary (researcher interpretation) themes from the individual studies were presented, and common categories across these themes were identified. These were iteratively refined through further discussion into comprehensive categories and sub-categories. Translating the studies into each other involved developing these categories, with detailed data from each study added and used to elucidate and elaborate these common categories. Table 3 gives an illustrative view of this process. The synthesis into higher order interpretations grounded in the findings of the individual studies – the focus of our discussion below– was developed iteratively through discussion and writing, using these categories and sub-categories as a starting point.

**Table 3:** Illustration of the process of aggregating themes and developing common categories

**Primary and secondary themes from the individual studies**	**Common categories identified and further developed through reciprocal translation**	
**Study 1**	**Category**	**Sub-category**
At one clinic patients accessing HIV treatment were sent to one nurse who had access to computer based records for HIV care	Health systems influence on service integration	Administration requirements with medical records, files, registers and monthly reporting specific to different programmes influences service integration
A clinic that initially integrated ART care into the work of all nurses experienced problems with recording of TB statistics and had to revert to more vertical delivery of care so that one nurse could concentrate on care of TB patients and collection of TB statistics		
**Study 2**		
Multiple registers for each programme require huge amounts of paperwork, which is one of the reasons why it is easier to have vertical programmes so each nurse has a specialty and the register to fill in for that specific condition.		
Because of the lack of resources, vertical approach simplifies and streamlines the large patient load (especially administration).		
**Study 3**		
Administrative demands on nurses to report on care provided and computer systems that require specific training and skills can support the separation of care.		

## Results

We identified three major categories in our synthesis of the factors that influenced integration of HIV care into all consultations: health systems issues; the way nurses perceived their work; and the influence of patients themselves on service organisation. These themes were identified in all three studies. The sub-categories within these three major categories had complex impacts on efforts to integrate HIV care: some factors facilitated integration of HIV care, others hindered integration, while still others tended to promote instead a more specialised or vertical approach to delivery of HIV care. Furthermore some factors appeared to have both facilitating and hindering effects on integration, depending on the setting.

### Health systems factors

Our findings suggested that a range of systems level factors hindered the integration of care at the primary care level. These factors included: the plethora of medical records, registers and monthly reports specific to each programme; the shortage of support personnel; and the infrastructure of many clinics with separate waiting areas and buildings for different services. The high workload in many clinics had complex effects on integration while the smaller size and staff complement of some clinics appeared to promote integration of care.

#### Administrative and reporting requirements

The administrative work required of clinic staff tended to reinforce the existing organisation of care and thus to hinder integration in a number of ways. For example, some programmes had specific forms for each consultation to ensure that important clinical information was elicited and recorded within the patient’s general file. In many clinics there were also separate medical files for HIV and other chronic diseases. In addition, many programmes had their own register in which the numbers of patients seen had to be recorded. These programme specific records tended to hinder efforts to integrate HIV care into all consultations within a clinic. For example in one clinic with many TB patients the former “TB nurse” struggled to cope with the addition of ART care on top of conducting the clinic’s TB data collection and statistical reporting. The clinic manager therefore decided to revert to the system of ART patients accessing care only from ART nurses. In another clinic, nurses in the ART programme had, in theory, access to a computer to directly enter consultation details for ART patients. However, only one consultation room had a computer and other nurses had to send all HIV positive patients to the ART nurse in that room for their consultation details to be captured. However, separate administrative processes and structures did not always hinder integration. In one large clinic patients collected ART files from a separate reception area because the main reception was too small yet they accessed care from any nurse as the nurses in that clinic had decided they would all be involved in all HIV care.

#### Staff shortages hindered integration

The shifting of clinical tasks to nurses requires adjustments in the roles of other supporting staff, such as pharmacists. Within the context of the STRETCH trial, a shortage of support staff tended to hinder the integration of HIV care. For example, provincial regulations at the time limited ART dispensing to pharmacists only. In clinics where there was no pharmacist, ARVs were dispensed monthly by hospital pharmacists and sent in patient-named packets to the clinics. Where there were, in addition, no pharmacy assistants at the clinic to issue these packets from the dispensary, nurses often responded by storing all the packets in one consultation room and having one nurse see, and issue drugs for, all patients on ART.

#### Physical infrastructure of clinics

The structure of clinics and the amount of space available also affected integration. Partly because of the history of separate service provision under different health programmes, a number of clinics had been built initially with different sections or had had extra sections built later, each with consulting and waiting rooms. Different programmes were allocated to separate sections and it was then difficult to integrate care. One STRETCH clinic, for instance, had two distinct parts within the building, each with separate waiting areas. Nurses referred to these as the ‘ART side’ and the ‘mainstream side’.

Some clinics had been provided with prefabricated structures by the ART programme or by donors to increase the number of consulting and waiting rooms available. This also hindered integration as it was easier to locate specific programmes, usually the ART services, in these prefabricated structures. Nurses were, however, aware of the stigma of locating ART services in these separate buildings. In one PHC clinic that was due to start providing ART and had received a prefabricated structure to provide more space, the clinic manager did not want to locate only ART services there for this reason. However, the presence of adequate physical infrastructure was not sufficient on its own to ensure integration. For example, there were a number of clinics with one large waiting area where integration of care did not take place because of other factors, such as nurses preferring to specialise or attempting to cope with large workloads by task allocation (as discussed below).

#### High nurse workload

The high workload of nurses in most of the clinics had complex effects on integration. In some cases, workload hindered integration because nurses felt unable to spend sufficient time with patients to provide comprehensive care. Nurses reported they did not have time to provide comprehensive care in all consultations. Although workloads varied, nurses were under pressure to achieve a quota of having 40 patient consultations per day. This led to a general pressure to work quickly and address only a limited number of issues in any one consultation. Many nurses were troubled by this, saying they knew they were not providing quality care and were not always able to integrate HIV care into their consultations.

Another strategy adopted by many clinics with large patient numbers, was to allocate specific tasks to one nurse in order to streamline the work. In some clinics, all patients who needed TB investigations were sent to one nurse for sputum collection, partly for reasons of infection control, but also to save time and ensure that one nurse could be responsible for collating results. In another clinic, with a large number of patients on ART, all patients needing routine blood tests were sent to one nurse on arrival at the clinic so that all bloods would be ready for collection before the arrival of the laboratory transport officer.

Nurses also reported that HIV positive patients had more potential illnesses, side effects and emotional aspects to consider and thus required longer and more complicated consultations than for other chronic diseases. This affected their willingness to provide integrated care. Some nurses were willing to be involved in it while others found it too overwhelming.

In other cases, nurses responded differently to the pressure of workload. At one clinic, the high workload of patients needing ART care had prompted all nurses to want to respond to their patients’ suffering. As a team, the nurses decided to reorganise delivery of care so that patients could access all HIV care from any nurse in that clinic. In another clinic, an ART nurse said that the high patient load meant she no longer had time to do all VCT and so all of the other nurses had become involved in VCT as well.

#### Clinic size

Large clinics with a large number of nurses tended to provide separate care under different programmes. The reasons underlying this were that, in most large clinics nurses would be allocated to one programme and be responsible for clinical care, reports and monthly statistics. Nurses could rotate through different programmes and gain comprehensive experience, but even so they tended to specialise in one programme and remain with that programme for extended periods of time. The possible extent of this separation is clear in one large clinic which had a “fast lane” for ART patients to go straight to the two ART nurses, without stopping at the clerks for their patient files. All other patients however could see any of the nurses for VCT and care before being referred to ART nurse for ARVs. However, large clinics did not always provide care separated into different programmes. One large clinic with 13 nurses provided fully integrated care. Patient files were uniform, there was one reception area and filing room, all waiting areas were integrated and patients were able to access any care from any nurse. This was reported to be due to the strong commitment of the clinic manager to integrated services.

In contrast, in clinics with only two or three nurses, each nurse had to be experienced in and responsible on a daily basis for caring for patients with health issues that spanned a number of programmes. In two clinics, each staffed by three professional nurses, patients could access integrated care from any nurse as there were many days when only two nurses were present due to training, meetings or leave. In other small clinics, however, not all consultations were fully integrated despite nurses having experience in all programmes. In one clinic, with three nurses, two of the nurses referred patients who needed HIV testing or care to the ART nurse, reportedly because these nurses did not speak the same language as many of their patients and did not feel they could provide HIV care to their patients.

### Nurses’ and managers’ beliefs and attitudes

Nurses often had a passion for developing specialist expertise in one area of care and also understood the importance of developing good nurse–patient relationships. Both of these factors tended to favour the separation of services by health condition (TB, HIV/AIDS) rather than integrated care. Nurse managers had mixed attitudes towards the integration of HIV care and this impacted significantly on efforts to integrate HIV care in their clinics.

#### Nurses’ preferences for particular programmes

Across primary care, nurses expressed a preference for working on a particular service or programme. They described this as an ‘interest’ or ‘passion’ for this service, such as TB, child or HIV care. This passion for a particular service tended to promote separate rather than integrated provision of HIV care:

I was saying that if people are not made to do this ARV thing comprehensively … because some of the people have got passion … like some of the sisters had passion for ARV, and the way they are with their clients you would say these people are friends. But when they were made to do child health some of them decided, no, I’m leaving this because I wanted to do this [ARV care]. This is my passion (STRETCH Nurse).

Although this desire to focus on a particular service was evident across primary care, there were also factors specific to HIV care. Providing HIV care, and ART in particular, often brought prestige and status to nurses. These nurses enjoyed being recognised by their community, with one clinic manager commenting that having ART in the clinic led to the community perceiving them as better nurses. In some cases, their colleagues felt that the prestige given to ART nurses, as well as their emotional attachment to ART care, made them reluctant to let other primary care nurses become involved. This issue led to sharp disagreement in one clinic between the ART and primary care nurses when HIV care was integrated into primary care services.

Some nurses noted that they did not have an interest in providing ART care. The commonest reasons for this were either that they would rather specialise in other areas of care or that they lacked the clinical confidence to provide ART care. This was related to the perceived complexity of providing ART and of delivering VCT. Similarly, ART nurses in some clinics raised concerns regarding whether all primary care nurses would be able to respond to this complexity and provide the same quality of care as they were able to. In a few clinics nurses reported that some of their colleagues did not want to provide ART care because they were afraid of HIV and stigmatised people living with this condition.

The effects of nurses’ desire to specialise were offset in some clinics by a broad based enthusiasm to be involved HIV care provision which, in turn, tended to promote more integrated care. For example, in some clinics all of the primary care nurses wanted to gain experience in ART care as part of their professional development. In one clinic, nurses described personal motivations for providing HIV care. Because HIV had impacted the lives of friends or family, they wanted to help alleviate the suffering that they saw in their community. In this clinic all nurses provided ART care and it was integrated into all consultations.

#### Nurse–patient relationships and continuity of care

Some nurses felt that it was easier to establish and maintain good nurse–patient relationships in settings where nurses were allocated to a particular programme rather than to a fully integrated service, in which patients were seen by any nurse. This concern for relationships with patients was expressed not only in relation to HIV care but also for other illnesses. Some nurses argued that external managers who had decided that services must be integrated did not appreciate the potential benefits of separate programmes in promoting what nurses perceived as good relationships with patients. In a health system that does not support patients choosing which nurse they would like to see, nurse specialisation does allow patients to see the same nurse over a period of time, and therefore to develop a relationship with that nurse and benefit from continuity of care.

#### Attitudes of clinic managers towards integration

How clinic based nurse managers and local area managers, who had oversight of more than one clinic, exercised their leadership also influenced the implementation of integration. Their views and preferences influenced integration, but this was also modulated by individual nurse preferences and administrative factors that characterised the vertical ART rollout.

In one large clinic, the manager was strongly supportive of integration of care within all consultations. Despite concerns raised by some staff members about the competence of all nurses in providing HIV care, patients in this clinic could access HIV care with any nurse in any consultation. In another clinic, a manager reported that, in order to implement her preference that HIV care be integrated into all consultations, she had to address significant resistance from some clinic nurses. One local area manager who supported integrated care explained some of the administrative obstacles:

…ARVs being handled like any other chronic disease. It must not be a special thing with special prescriptions and programmes on the computer, with special [ART] files and forms and those kinds of things. I want it handled like hypertension and diabetes (Local area manager).

Other managers were not supportive of full integration of HIV care into every consultation. One manager in an ART STRETCH clinic felt that it was beneficial for nurses to have a specialised focus in one area of primary care. Another clinic manager was not confident in providing HIV care herself and was not willing to integrate HIV care into all her consultations.

### Patient preferences regarding delivery of care

Patient preferences had conflicting influences on the integration of HIV care. Patients preferred to receive services from a nurse with expertise in HIV care, and whom they knew and trusted. However, they also had concerns about the stigma of accessing HIV care in separate services.

#### Nurse–patient relationships

Patients preferred to be seen by a particular nurse either because they had established a relationship with that nurse or because they perceived the nurse to have developed expertise in ART care. This created pressure for separate care rather than integrated care:

Again we want to have our own nurse. Sometimes we experience personal problems that we would like to discuss with our nurse but it is not easy if today you are seen by this one and next time is that one (Patient).

One group of patients reported concern when ‘their’ ART nurse left and they were moved from the ART side of the clinic and asked to wait with other patients and be seen by other nurses. They felt that these other nurses needed more training and did not have the same level of expertise as their ART nurse. Many ART patients were, or had been, very ill and were therefore reluctant to be seen by nurses other than the nurse they knew well and trusted. In one clinic, patients reportedly preferred waiting in their own area to see the ART nurse as this allowed them to share information with other patients on ART and to prevent other patients from pushing in. In one ART STRETCH clinic, nurses reported that ART patients had resisted efforts to integrate services by continuing to visit “their nurse”.

#### The stigma of separate HIV services

Despite patients’ desire to see a nurse with experience in ART care, they were also concerned about the stigma of accessing care through a specialised service. Many patients disliked the stigma of being identified in their community as ‘ART patients’ through their use of a separate section of the clinic, and through having their ‘own’ nurses. They claimed that they felt less HIV-related stigma when they were called from the main waiting room like any other patient. Even so, they continued to express a preference for seeing the nurse with whom they were familiar:

We don’t have a problem waiting with everyone but we want our files separated and our nurse should just call our names and we go to our specific room” (Patient).

Similarly, nurses in a number of clinics expressed concern that some patients resisted accessing treatment delivered through specialized HIV and ART nurses or specific waiting areas because of their fear of stigma. Nurses at one clinic reported that the number of patients tested for HIV increased markedly when all nurses were involved in providing HIV testing as well as other HIV care. Another ART clinic reportedly received increasing numbers of patients when they changed from having a separate ART waiting area to an integrated waiting area.

## Discussion and conclusion

This synthesis of findings has identified three main categories of factors that affect efforts to integrate HIV care into primary care services. These are the characteristics of the health system itself; nurses’ and managers’ preferences regarding how to deliver care; and patients’ preferences for how they would like to receive care. Factors hindering the integration of HIV care into all consultations included the existing organisation of clinical records and reporting; high workloads and shortages of support staff; and the structure and organisation of existing clinic buildings. Factors that promoted the delivery of HIV care as a separate programme included nurses’ preferences to develop expertise and specialise in particular areas of care as well as the value that nurses and patients placed on nurse–patient relationships. On the other hand, factors that promoted the integration of HIV care into all consultations included: a widespread concern amongst nurses to become involved in dealing with the HIV pandemic; and nurse and patient concerns about the stigma of separate HIV services.

Our findings show that even when efforts are made to integrate HIV care, the complex interaction of these three factors results in a variety of models of delivery of HIV care depending on local context –such as clinic size. These models varied on a spectrum from a fully integrated service, where patients accessed HIV care from any nurse, to a more separate delivery of care, with patients accessing care from an ART nurse. This variability in the outcome of efforts to integrate HIV care during the STRETCH trial –a complex intervention of task shifting – may have been one of the contributorsx to a lack of significant improvement at intervention clinics in survival of patients needing ART [35]. A quantitative study of integration conducted during the trial showed that patient survival was significantly improved in clinics with high integration scores [36].

These findings also suggest that as long as nurses are expected to manage high numbers of patients each day in primary care, HIV care is unlikely to be successfully integrated into service delivery. This finding is especially critical in countries like South Africa where criteria for ART eligibility have been widened and large numbers of people are now eligible for ART. Attention therefore needs to be given to the adequate resourcing and staffing of primary care services. A recent technical brief on integrated care from the WHO argues clearly that integrated care is not a solution to a shortage of health care workers [37]. Attention also needs to be paid to other areas of health system functioning [16,17]. The integration of HIV care into the governance, finance and planning of services would ensure that adequate staffing levels, and even new clinic design and the renovation of clinics, are planned and financed adequately and are aimed at the support of integrated service delivery. Full integration of administration, monitoring and evaluation of different programmes may not be possible or even desirable. However, serious efforts need to be made to have clear policies on the integration of services [38], to simplify and integrate patient records and registers and to provide supervision of and training in integrated care to support primary care nurses [7]. If these other areas of health system functioning are not coordinated in order to support integrated service delivery, nurses will continue to be frustrated in their attempts to provide integrated care.

It is also apparent from these findings that the model of integration of HIV care into primary care that is most preferred by patients and nurses may not always be the full integration of HIV care into all consultations. Some degree of specialisation of nurses, where they can be well trained and develop expertise may be desirable and this has been noted in a recent study of the barriers to integration of sexual and reproductive health services [38]. Both patients and nurses expressed their appreciation of the opportunity for nurses to develop expertise and specialise in certain areas of care, and noted the benefits of this for promoting nurse–patient relationships. Even where nurses and patients expressed concern about the stigma of separate HIV services, there was a certain tension with the wish to be seen by nurses with expertise and with whom they were familiar. A recent systematic review of the outcomes of integration of services into primary care noted evidence that integration of some services can lead to decreased utilisation and the need for studies of outcomes to document patient views of the desirability of integration [15]. The recent WHO technical brief on integration also comments that integration does not mean all services must be provided in one package but is rather about delivering the ‘right care’ in the ‘right place’, which is easy to access and achieves the desired results [37].

It may also be that the ‘best model’ for delivering HIV care in primary care varies according to local circumstances. This synthesis showed that the spectrum of integration within clinics in the STRETCH trial and the models of care that developed were at least in part influenced by the preferences of local staff, patients and managers. While this study does not assess the impacts of different models of integration of HIV care on patient outcomes, it does suggest that such integration is a process that takes time, needs support and needs to be responsive and adaptable to local conditions. The process evaluation of the STRETCH intervention also noted the positive impact of local support teams and external facilitators and the importance of mentoring in supporting task shifting [30].

The meta-ethnographic approach used in this study has allowed us to integrate findings from multiple studies and bring depth of insight to a number of issues surrounding the integration of HIV care into primary care services. Additional advantages of our approach are the team’s deep knowledge of the study setting and their access to the full datasets for the studies, rather than the published papers only. One example of the usefulness of this rich dataset is that many of the factors identified had complex and even conflicting effects on integration in different contexts – these nuances may not have been identified through a review of less detailed data available from published papers. Although this study focussed on integration of HIV care, some of the findings are similar to those of a study on integration of sexual and reproductive health services in South African publicly funded clinics [38], and so are likely to be generalizable to other areas of integration in service delivery. One of the limitations is that the studies were not designed together and methods and theoretical frameworks differed. Another potential limitation is that the nature of participant observations is that they may have been affected by the researchers involvement in the trial.

Based on our conclusions, we make three recommendations: firstly, the design, implementation and management of service integration should engage with and account for local variability and the active influence of nurses and patients, through closer, more dynamic approaches to management. Secondly, future research should explore which configurations of integration are best suited to different settings and should in particular explore patient and nurse preferences for integrated versus separate services, and how nurse–patient relationships can be maintained and supported within different models of care. Thirdly, the integration of management, administration, financing, planning and monitoring systems may help to support efforts to tailor the integration of HIV care into primary care at clinic level and further work in this area is needed.

## Ethical approval

Permission to conduct these studies was obtained from the Head of the Free State Department of Health. Ethical approval of the protocols of these studies was obtained from the Human Research Ethics Committees of the London School of Hygiene and Tropical Medicine and the Faculty of Health Sciences of the University of Cape Town and the University of the Free State.

## Abbreviations

AIDS: Acquired immune deficiency syndrome; ART: Antiretroviral therapy; ARVs: Antiretrovirals; HIV: Human immunodeficiency syndrome; TB: Tuberculosis; VCT: Voluntary counselling and testing; STRETCH: Streamlining tasks and roles to expand treatment and care for HIV.

## Competing interests

The authors declare they have no competing interests.

## Authors’ contributions

KU was involved in developing the initial concept, data collection and synthesis and writing the manuscript. DG and AG were involved in developing the initial concept, data collection, analysis and synthesis and writing the manuscript. SL and CC were involved in the initial concept, data collection and analysis and reviewing the manuscript. All authors read and approved the final manuscript.

## Pre-publication history

The pre-publication history for this paper can be accessed here:

http://www.biomedcentral.com/1472-6963/13/171/prepub
